# Use of N-acetylcysteine as treatment adjuvant regulates immune response in visceral leishmaniasis: Pilot clinical trial and *in vitro* experiments

**DOI:** 10.3389/fcimb.2022.1045668

**Published:** 2022-11-24

**Authors:** Lucas Sousa Magalhães, Enaldo Vieira Melo, Nayra Prata Damascena, Adriana Cardoso Batista Albuquerque, Camilla Natália Oliveira Santos, Mônica Cardozo Rebouças, Mariana de Oliveira Bezerra, Ricardo Louzada da Silva, Fabricia Alvisi de Oliveira, Priscila Lima Santos, João Santana da Silva, Michael Wheeler Lipscomb, Ângela Maria da Silva, Amélia Ribeiro de Jesus, Roque Pacheco de Almeida

**Affiliations:** ^1^ Laboratory of Immunology and Molecular Biology, University Hospital, Federal University of Sergipe, Aracaju, Brazil; ^2^ Health Sciences Graduate Program, Federal University of Sergipe, Aracaju, Brazil; ^3^ Sector of Parasitology and Pathology, Biological and Health Sciences Institute, Federal University of Alagoas, Maceió, Brazil; ^4^ Department of Medicine, University Hospital-Empresa Brasileira de Serviços Hospitalares (EBSERH), Federal University of Sergipe, Aracaju, Brazil; ^5^ Department of Health Education, Federal University of Sergipe, Lagarto, Brazil; ^6^ Department of Biochemistry and Immunology, Ribeirão Preto Medical School, University of São Paulo, Ribeirão Preto, Brazil; ^7^ Department of Pharmacology, University of Minnesota, Minneapolis, MN, United States; ^8^ Immunology Institute of Investigation (iii), National Institute of Science and Technology (INCT), Brazilian Research and Technology Council (CNPq), São Paulo, Brazil

**Keywords:** visceral leishmaniasis, N-acetyl-l-cysteine, adjuvant chemotherapy, meglumine antimoniate, drug therapy

## Abstract

This investigation aimed to assess the effect of N-acetylcysteine (NAC) as an adjuvant treatment to alleviate visceral leishmaniasis (VL). The present work includes both blinded randomized clinical intervention and experimental *in vitro* studies. The clinical trial included 60 patients with VL randomly allocated into two groups: a test group (n = 30) treated with meglumine antimoniate plus NAC (SbV + NAC) and a control group (n = 30) treated with meglumine antimoniate only (SbV). The primary outcome was clinical cure (absence of fever, spleen and liver sizes reduction, and hematological improvement) in 180 days. The cure rate did not differ between the groups; both groups had similar results in all readout indices. The immunological parameters of the patients treated with SbV + NAC showed higher sCD40L in sera during treatment, and the levels of sCD40L were negatively correlated with Interleukin-10 (IL-10) serum levels. In addition, data estimation showed a negative correlation between the sCD40L levels and the spleen size in patients with VL. For the *in vitro* experiments, peripheral blood mononuclear cells (PBMCs) or PBMC-derived macrophages from healthy donors were exposed to soluble *Leishmania* antigen (SLA) or infected with stationary promastigotes of *Leishmania infantum* in the presence or absence of NAC. Results revealed that NAC treatment of SLA-stimulated PBMCs reduces the frequency of monocytes producing IL-10 and lowers the frequency of CD4+ and CD8+ T cells expressing (pro-)inflammatory cytokines. Together, these results suggest that NAC treatment may modulate the immune response in patients with VL, thus warranting additional investigations to support its case use as an adjuvant to antimony therapy for VL.

## 1 Introduction

Visceral leishmaniasis (VL) is an infectious disease caused by digenetic parasites of the species *Leishmania (Leishmania) infantum* or *L. donovani*. If left untreated, the disease can be fatal. Further compounding, the challenge is that the disease largely affects neglected populations—those without adequate healthcare and infrastructure support (Burza et al., 2018).

It is well established that VL is a disease caused by dysregulation of immune response. Several studies demonstrate that *Leishmania* parasites actively induce alterations in immune cell responses. This is most notable during parasite internalization by macrophages, which includes the inability to adequately clear the pathogen load (Liévin-Le Moal and Loiseau, 2016). Results demonstrate that patients with better disease progression have antigen-specific immune responses; this is chiefly based on an early action of IL-12 and Interferon‐γ (IFN-γ) to promote immune effector responses (Dayakar et al., 2019). However, patients with VL with severe disease burden have exacerbated inflammatory response, a phenomenon known as cytokine storm, a mixture of inflammatory and anti-inflammatory cytokines from innate and adaptive immune responses (Bhor et al., 2021).

Pentavalent antimonial compounds are the major drugs used to treat VL. The drug is relatively low in cost to manufacture and can induce robust pro-inflammatory responses (Aruleba et al., 2020). However, treatment can have adverse side effects, which can include cardiotoxicity, hepatotoxicity, nephrotoxicity, and pancreatitis (Kumari et al., 2021). Moreover, there are alternative drugs, such as Amphotericin B, that are efficient in curing VL in administered patients. However, the immediate drawbacks in use are the high cost and the associated toxicity (Kumari et al., 2022). Similarly, other drugs available for treatment of VL are miltefosine and paromomycin that also have good cure rates in patients with VL. Unfortunately, accessibility is limited in afflicted countries, and there are associated reports of treatment failure (Chakravarty and Sundar, 2019; Musa et al., 2022).

N-acetylcysteine (NAC) is a metabolite of L-cysteine amino acid widely used as antioxidant treatment (Dodd et al., 2008). NAC administration results in (1) increased levels of glutathione, (2) scavenging of free radicals like hydrogen peroxide, and (3) reduction of disulfide compounds (Pedre et al., 2021). Furthermore, NAC has been shown to regulate immune responses especially by downregulating Nuclear factor-κB (NF-κB) activation in dendritic cells (Giordani et al., 2002) and diminishing B cell activation (Bonnaure and Néron, 2014). Numerous works have demonstrated the protective effects of NAC against infectious diseases (Amaral et al., 2016; Gao et al., 2017).

Therefore, knowing that VL disease is associated with a dysregulation in immunity and that NAC treatment directly regulates immune responses, these studies aimed to evaluate whether NAC treatment as an adjuvant to antimony treatment in VL could course correct VL-induced deficiencies in immunity to improve clinical outcomes. Our data demonstrate that NAC has immune modulatory effect in *in vitro* experiments involving either T cells or monocytes and alters production of immunological biomarker as adjuvant during treatment of patients with VL. These data give evidence that NAC could be further explored as adjuvant treatment to patients with VL.

## 2 Material and methods

### 2.1 Study design and ethics statement

This work has two interconnected parts: (1) a pilot blinded and randomized clinical intervention study, with patients with VL treated with antimony plus NAC compared with patients with VL treated with antimony; and (2) an *in vitro* experimental study to evaluate the effect of antimony plus NAC in immunomodulation of monocytes, macrophages, and T-cell effector responses.

All patients and healthy donors included in the studies were recruited and signed informed consent for participations in the studies. The Research Ethical Committee from the Federal University of Sergipe approved this study (advice number 1.353.887). The clinical trial pilot was registered in clinicaltrials.gov database (NCT01138956).

### 2.2 Pilot blinded and randomized clinical trial study to evaluate the effect of N-acetylcysteine as adjuvant therapy to antimony for VL treatment

All patients included in this study were invited to participate after VL diagnostics. All patients or their legal guardians signed an informed consent. Patients were consecutively recruited between 2008 and 2010 at the University Hospital of the Federal University of Sergipe in northwestern Brazil, which is an endemic area of VL, where approximately 64 new cases of VL are reported per year (Campos et al., 2017). On the basis of the number of new patients with VL, the participation of 60 patients was estimated for this clinical trial study, adopting a significance level of 0.05 and a power of 0.8.

VL diagnosis was confirmed with positive serology to rK39 antigen and positive culture from bone marrow. The clinical criteria that are utilized included fever, liver and spleen (increased) sizes, diarrhea, epistaxis, jaundice, cough, and signs of anemia. Additional laboratory criteria included anemia, leukopenia, thrombocytopenia, hypoalbuminemia, and hyperglobulinemia. Patients with other chronic or acute diseases, who were pregnant, had severe neutropenia, or who used immunosuppressors were excluded from the study’s participation pool. All patients were HIV-negative and showed no cardiac alterations in electrocardiogram. The protocol used in the study does not present risks to the patients.

Two groups were defined to the study, and all patients included were randomized using a computer randomization table: (1) test group (n = 30): received meglumine antimoniate or Glucantime^®^ as standardized plus NAC, three doses of 600 mg/day (a dose every 8 h), oral route, for the same period (SbV + NAC); (2) control group (n = 30): received Glucantime^®^ (SbV) as standardized by Brazilian Ministry of Health, 20 mg/kg/day for 20 days, intravenously.

The medical team assessing outcomes was blinded during patient’s follow-up. Patients included in control group did not receive placebo treatment. A follow-up of clinical and laboratorial change over time was performed by the clinical staff for 180 days. The data included in this study were at the following time points: D0, before treatment; D10 and D20, during treatment; and D30, after treatment. Blood samples were obtained in these time points and used for the laboratorial tests: blood count, dosage of proteins, and alanine and aspartate aminotransferases; and for serum cytokines, measurement at D0, D15, and D30.

The primary outcome was defined as the cure of VL 180 days after treatment and based on the following key readout indices: reduction of liver and spleen sizes, absence of fever, weight gain, and improvement of leucocytes and erythrocytes numbers.

#### 2.2.1 Parasite load

Parasite load was evaluated in blood sample of patients during treatment according to previous published works (Schulz et al., 2003; de Oliveira et al., 2013).

#### 2.2.2 Cytokines quantification

Cytokines in sera were measured in three different time points: D0, D15, and D30. The serum levels of Tumor Necrosis Factor-α (TNF-α), IL-12p40, IL-10, and sCD40L were measured using Luminex multiplex assay, according to manufacturer’s recommendations (MILLIPLEX MAP Human Magnetic Bead Panel) (Merck Millipore, USA), read in a Luminex 200, and analyzed by a Milliplex Analyst software.

### 2.3 *In vitro* study to evaluate the effect of NAC plus antimony in *L. infantum* infection

#### 2.3.1 Obtention of PBMCs

Peripheral blood mononuclear cells (PBMCs) were obtained from heparinized whole blood of the healthy donors following previously published approach(es) (Barrios et al., 2019). Briefly, PBMCs were isolated by density gradient using Histopaque^®^ 1077 (Sigma-Aldrich, USA). Cells were then washed and resuspended with supplemented RPMI 1640 media (10% of inactivated fetal bovine serum plus 1% of penicillin/streptomycin) (Gibco, USA).

#### 2.3.2 N-acetylcysteine concentration

To development of *in vitro* experiments using human cells, the concentration of NAC was determined in 10 mM on the basis of the previously published data in literature (Giodani et al., 2002; Cruz et al., 2008).

#### 2.3.3 PBMC stimulation, treatment, and flow cytometry protocol

PBMC assays were based on the previously published methods (Santos et al., 2017). Briefly, PBMCs were seeded in 48-well plates at a density of 1 × 10^6^/well. Cells were treated under different conditions: (a) unstimulated; (b) stimulated with Soluble *Leishmania* Antigen (SLA; 1 mg/ml); (c) stimulated with SLA (1 mg/ml) plus antimoniate meglumine (20 µg/ml); (d) stimulated with SLA (1 mg/ml) plus 10 mM of NAC (Sigma-Aldrich, USA) or stimulated with SLA (1 mg/ml) plus antimoniate meglumine (20 µg/ml) plus 10 mM of NAC. After 6 h of incubation, Brefeldin A (BD Biosciences, USA) was added, and the cells were then seeded for an additional 12 h. The plates were washed with 1× PBS prior to blocking with 2% fetal bovine serum and 2% of fetal goat sera. After 30 min, plates were washed and incubated with the following fluorescent antibodies for cell surface markers: CD3 PE-Cy7 and CD4 V500, or CD14 PE-Cy5 and CD40L V500 (BD Pharmigen, USA). After 20 min, cells were washed, permeabilized, and incubated with the following fluorescent antibodies for cytokines for intracellular detection: IL-2 BV421, TNF-α Alexa Fluor 488, and IFN-γ APC or IL-10–APC and TNF-α PE (BD Pharmigen, USA). The cells were then washed and resuspended in 1× PBS. A BD FACSCanto II (BD Biosciences, USA) flow cytometry was used to acquire a minimum of 50,000 cells. Datasets were analyzed using FlowJo software (BD Biosciences, USA).

#### 2.3.4 Transcription factor expression and qPCR

RNA was isolated from PBMCs using TRIzol (Thermo Fisher Scientific, USA) following the manufacturer’s recommended protocol. Total RNA was used for qPCR assays using TaqMan probes (Thermo Fisher Scientific, USA) to TBX21, GATA3, and FOXP3 genes. Amplification was performed using a 7500 Fast Real-Time PCR system (Thermo Fisher Scientific, USA).

#### 2.3.5 Parasite culture

One strain of *L. infantum* parasite was used in the macrophage infection assays (LVHSE17, isolated from patient not included in the studies). *L. infantum* strain was cultivated at 24°C in B.O.D. incubator in Schneider’s Insect Medium (Gibco, USA) supplemented with 10% inactivated bovine fetal sera and 1% penicillin/streptomycin (Gibco, USA).

*Macrophage infection and treatment with NAC plus antimony*

Macrophage differentiation, infection, and treatment followed prior published works (Barrios et al., 2019). Briefly, healthy donors’ PBMCs were seeded in Nunc LabTek™ chamber slides (Thermo Scientific, USA) at a density of 5 × 10^5^ cell per well. After 2 h of incubation, non-adherent cells were removed by washing. Adherent monocytes were differentiated for 6 days in an incubator (at 5% CO_2_ and 37°C). Next, macrophages (1 × 10^5^ per well) were co-cultured with stationary promastigotes of *L. infantum* at a 10:1 ratio for 2 h. Next, plates were washed to remove the non-internalized parasites and were incubated for 2, 24, and 72 h under different conditions as afore-described. Supernatants were removed, and cells were fixed with absolute methanol prior to staining with rapid panoptic (LaborClin, Brazil). A number of amastigotes and infected macrophages were determined at different time points by microscopy, and the parasite index was calculated (i.e., number of infected macrophages × amastigotes/100 macrophages). All experiments were performed in triplicate.

#### 2.3.6 Parasite viability analysis after NAC treatment

The ability of NAC in killing *L. infantum* parasites was tested using a dose–response curve, as previously published (Magalhães et al., 2018). Briefly, mid-log phase promastigotes of *L. infantum* isolate used in the experiments were washed (described above) and exposed to crescent concentrations of NAC (0.1 to 80 mM) in Schneider Insect’s Medium for 48 h. Parasites’ viability was determined by counting motile promastigotes in a Neubauer chamber.

### 2.4 Statistical analysis

D’Agostino-Pearson normality test was used for testing the Gaussian distribution. Differences among groups were considered statistically significant when p < 0.05. Friedman test followed by Dunn’s test was used for multiple comparisons. Wilcoxon test was used for the simple paired comparison, and Mann–Whitney test was used for simple unpaired comparison. R software was used in the construction of heatmaps (Heatmaply and ComplexHeatmap packages). Graphs were made using GraphPad Prism. Correlations were tested using Pearson’s r. Bootstrapping method was used to resample data and test correlation between cytokine levels and clinical parameters. Effect sizes of the differences between groups were calculated in the pilot clinical trial (Martins et al., 2021). Using IBM^®^ SPSS Statistics, we calculated one-way multivariate analysis of variance (MANOVA) to compare age and group effects in the pilot clinical trial, and effect size r of Mann–Whitney was used to compare various quantitative variables at the end of the treatment.

## 3 Results

### 3.1 Treatment of patients with VL with antimony + NAC increased sCD40L and decreased IL-10 levels in sera

**Figure 1:**
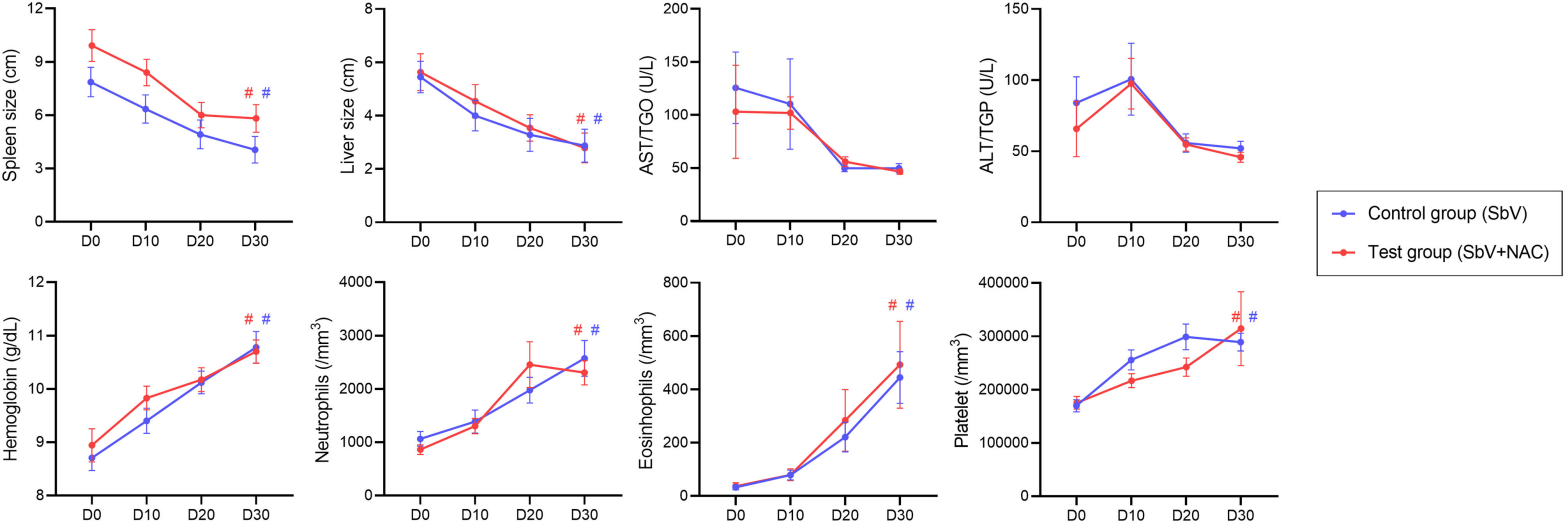
Clinical and laboratorial parameters measured from patients in the pilot clinical trial study. Data are obtained before (D0), during (D10 and D20), and after treatment (D30) of the control group (patients treated only with meglumine antimoniate for 28 days; SbV, blue) and the test group (patients treated with meglumine antimoniate plus NAC for 20 days; SbV + NAC, red). Dots and their lines represent mean ± SEM of patients in the group (n = 30). Kruskal–Wallis test followed by Dunn’s test was used for comparisons between groups. #p < 0.001 (internal comparison between D30 and D0 for each group).

Sixty-eight patients were diagnosed with VL. Sixty patients were selected to participate of the clinical trial. Thirty patients were randomly allocated in the test group that administered meglumine antimoniate plus NAC treatment (SbV + NAC). For the control group, 30 (separate) patients received standard meglumine antimoniate treatment (SbV group), without the NAC treatment (**Supplementary Figure 1**). A summary of clinical and laboratory data of patients enrolled in the studies is presented in **Supplementary Table 1**. The clinical and laboratorial parameters were evaluated before treatment (D0, before treatment), during treatment (D10 and D20, days 10 of 20 after treatment initiation), and after completion of treatment regimen (D30, 28 days after treatment initiation). **Figure 1** shows no differences between the SbV group and the SbV + NAC group in the clinical and laboratorial parameters: spleen and liver sizes, hepatic enzymes, hemoglobin levels, platelets, neutrophils, or eosinophil counting. Beyond that, data from 32 patients (16 from each group) were used to make a heatmap using the major clinical and laboratorial parameters. Results show that there is no significant difference in patterns of responses between the groups independent of the treatment stage and parameter analyzed (**Supplementary Figure 2**).

**Figure 2:**
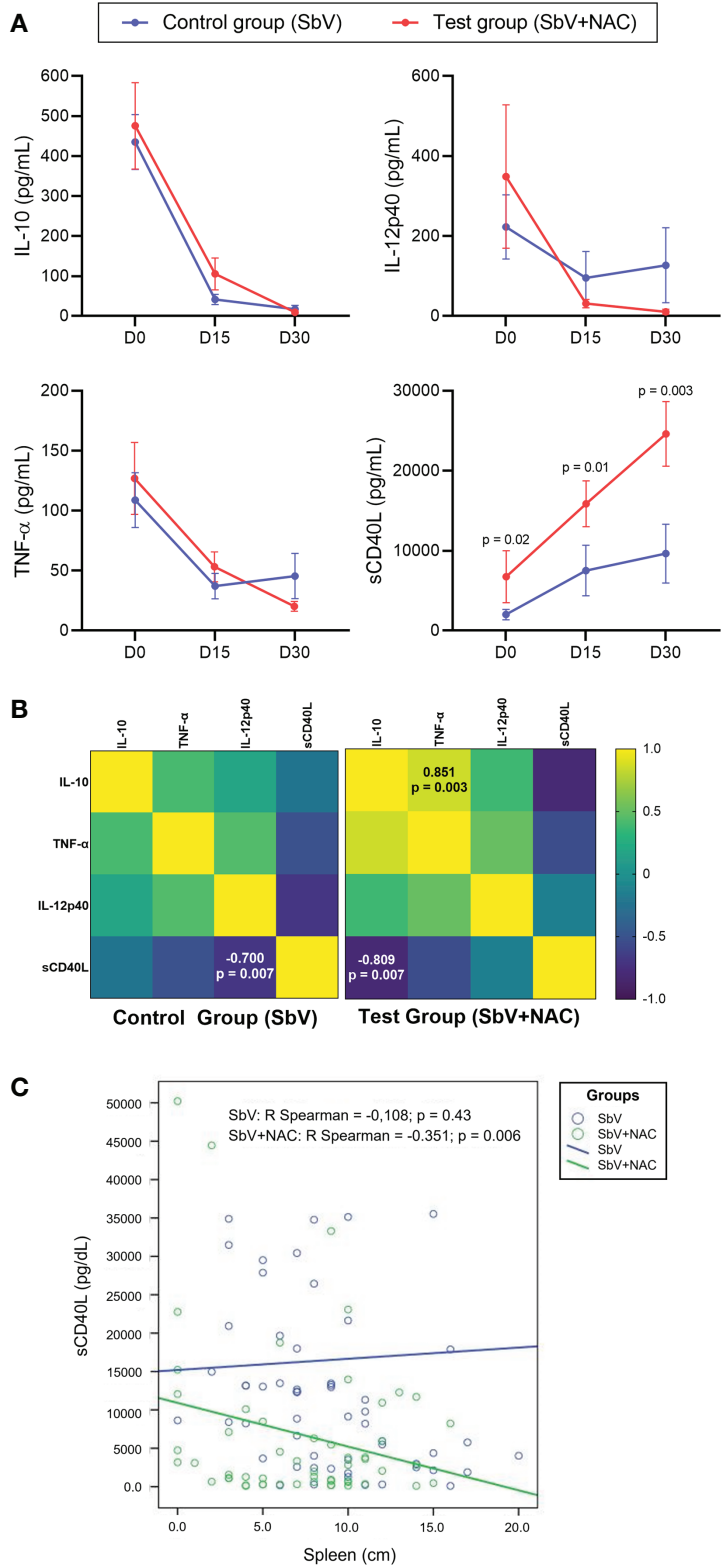
Immunological markers quantified in sera of patients included in pilot clinical trial study. Data are obtained before (D0), during (D15), and after treatment (D30) of the control group (patients treated only with meglumine antimoniate for 28 days; SbV, blue) and the test group (patients treated with meglumine antimoniate plus NAC for 20 days; SbV + NAC, red). **(A)** Dots and their lines represent mean ± SEM of patients in the group (n = 14 for SbV and n = 10 for NAC). Kruskal–Wallis test followed by Dunn’s test was used for comparisons comparison between groups. **(B)** Correlation among levels of cytokines measured during treatment (D15) in patients of different groups was made using the Spearman correlation test. **(C)** For correlation between the sCD40L levels and the spleen size, data are resampled using bootstrapping. Dots represent resampled data, and lines represent correlation.

To evaluate the immunological aspects from these patients, serum levels of IL-10, sCD40L, TNF-α, and IL-12p40 were measured at D0, D15, and D30. Observed results revealed that the test group, treated with SbV + NAC, had higher levels of sCD40L in all days evaluated when compared with the controls (**Figure 2A**). A significant difference between groups was observed when comparing the levels of sCD40L. Correlation analysis of sera during treatment shows that sCD40L of patients treated with SbV + NAC had a strong negative correlation with IL-10 levels (R = −0.809, p = 0.007) (**Figure 2B**). Moreover, IL-10 levels in sera during treatment of patients in the SbV + NAC group had a positive correlation with TNF-α (R = 0.851, p = 0.003). The sera from patients of the control group (SbV) show only a negative correlation between IL-12p40 and sCD40L (R = −0.700, p = 0.007). **Supplementary Table 2** shows a comparison of clinical, laboratorial, and immunological parameters between the groups, as well as the effect sizes at D30. There is a significant difference between groups, and a strong effect size was observed when comparing levels of sCD40L. The cure rate is the same for both groups (100% of cure). There are no differences in clinical or laboratorial parameters between groups.

We also tested the association of sCD40L produced and released during treatment and the major clinical and laboratorial aspects evaluated in the study. Initial analysis shows the absence of significant correlation among sCD40L levels and the clinical and laboratorial aspects in SbV + NAC group or SbV group (**Supplementary Figure 3**). Using the bootstrapping procedure to resample data and to make correlations, a negative correlation between the spleen size and the levels of sCD40L in the SbV + NAC group (R = −0.351, p = 0.006), but not in the control SbV group, was determined (**Figure 2C**).

There was a negative correlation between sCD40L in sera and the parasite load in patients of the SbV + NAC group (R = −0.442, p = 0.007), but not observed in SbV group (R = −0.104, p = 0.6) (data not shown in figure). Beyond that, one-way MANOVA was conducted to determine a difference between SbV + NAC groups and controls of sCD40L on days 0, 15, and 30 (**Supplementary Table 3**). Notably, there was a significant difference in the rank of sCD40L in the group (F = 5.184, p = 0.008; Wilk’s lambda = 0.425, partial eta squared = 0.425, observed power = 0.867). Furthermore, there was a significant effect on day 0 (F = 8.552, partial eta squared = 0.271, observed power = 0.800), on day 15 (F = 11,489, partial eta squared = 0.333, observed power = 0.901), and day 30 (F = 14,788, partial eta squared = 0.391, observed power =0.957). There was no significant difference in hemoglobin, amylase, platelets, and alanine aminotransferase (ALT/TGP) levels (data not shown).

### 3.2 *In vitro* experiments confirm that N-acetylcysteine reduces IL-10 production in monocytes and the activation of T CD4+ and CD8+ cells

**Figure 3:**
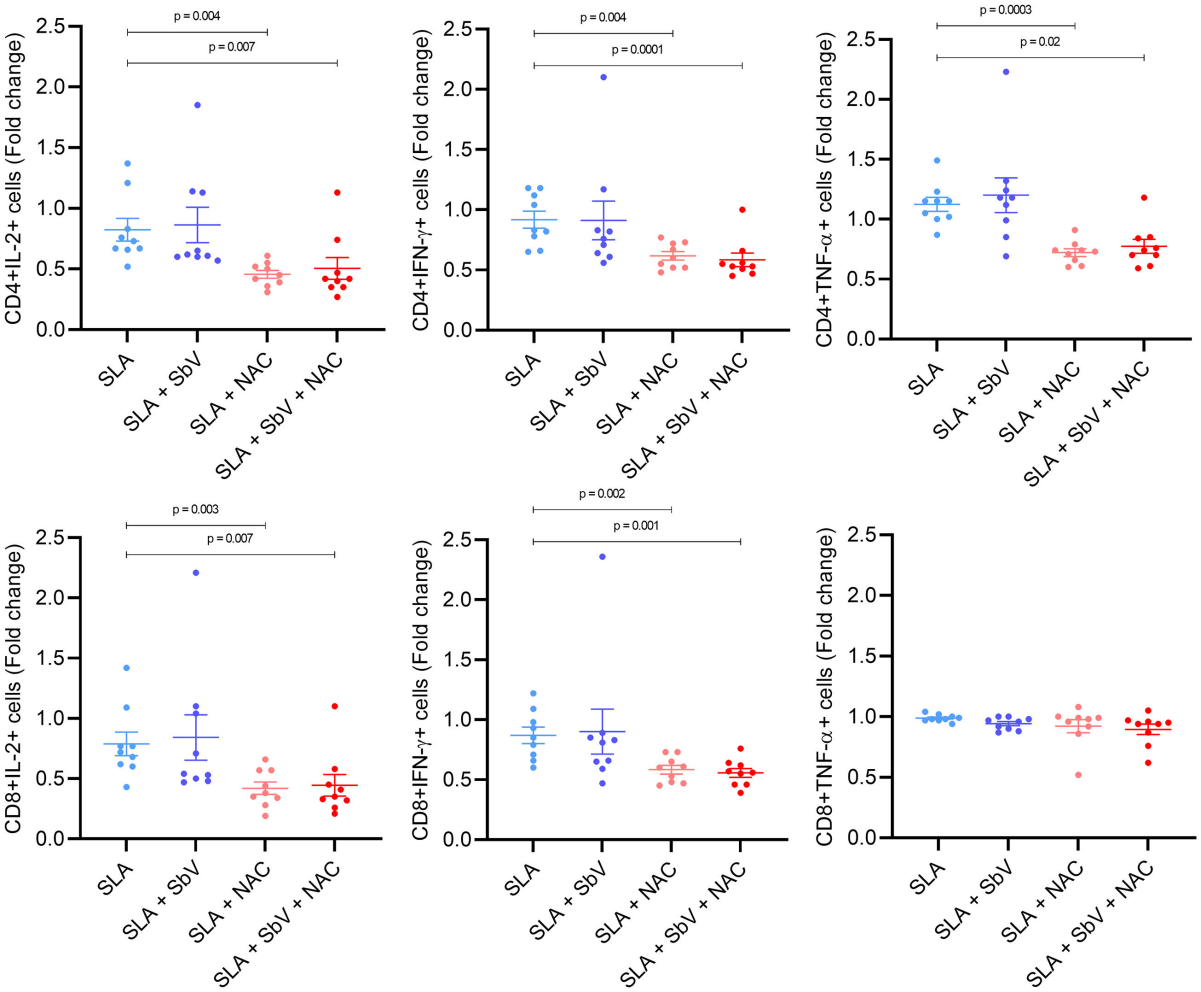
Frequency of CD4+ and CD8+ T cells producing IL-2, IFN-γ, or TNF-α in PBMCs stimulated with SLA and treated or not with SbV, NAC, and SbV + NAC. Graphs represent a fold change from unstimulated cells. Dots represent each healthy donor (n = 9), and lines represent mean ± SEM. Friedman test followed by Dunn’s test was used for comparisons.

**Figure 4:**
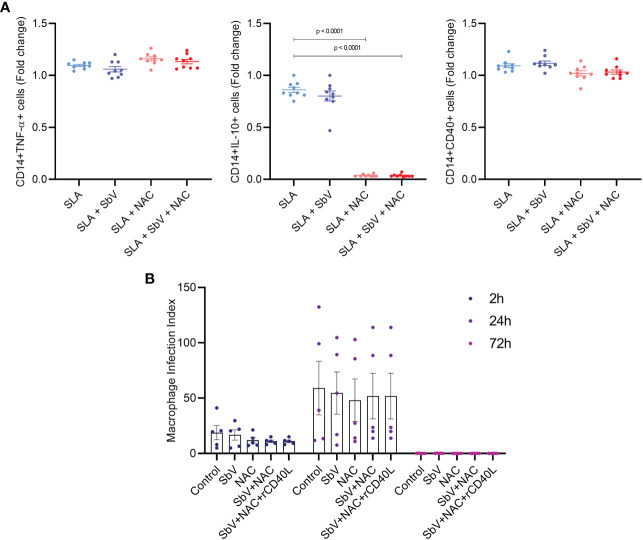
**(A)** Frequency of CD14+ monocytes producing TNF-α, IL-10+, CD40+ in PBMCs stimulated with SLA and treated or not with SbV, NAC, and SbV + NAC and obtained by flow cytometry. Graphs represent a fold change from unstimulated cells. Dots represent each healthy donor (n = 9), and lines represent mean ± SEM. **(B)** Macrophage infection index obtained from counting macrophages differentiated from PBMC and infected with *L. infantum* isolate (n = 5, in duplicate). Friedman test followed by Dunn’s test was used for comparisons.

The effect of NAC treatment in SLA-stimulated PBMCs was evaluated in monocyte cytokine production and T-cell functioning. The addition of NAC significantly reduced the frequency of monocytes CD14+IL-10+ and not changed TNF-α+ cells frequency when compared to PBMCs treated only with meglumine antimoniate, leading to a higher TNF-α/IL-10 ratio in NAC-treated cells (**Figure 3**). In addition, NAC treatment of PBMC reduced the frequency of CD4+ T cells producing IL-2, TNF-α, and IFN-γ, either in the presence or the absence of meglumine antimoniate (**Figure 4**). Furthermore, results reveal a similar reduction of CD8+ T cells producing IL-2 or IFN-γ. Moreover, no effects were observed between the cells treated only with meglumine antimoniate, when compared to cells stimulated or not with SLA. The results of SLA-stimulated PBMCs treated with NAC reveal no significant reduction of GATA3 and FOXP3 or/and increased TBX21 transcripts (**Supplementary Figure 4**).

To evaluate the effect of NAC in the macrophage microbicidal activity, macrophages were infected with *L. infantum* and treated with NAC and/or SbV. Treatment of macrophages with NAC does not change the number of infected cells or parasite load in these cells. This is represented by unaltered parasite index at any time point (**Figure 3**). It was also tested whether NAC could reduce viability of *L. infantum* parasites. The data show that treatment of promastigotes with increasing concentrations of NAC for 48 h does not reduce parasites’ viability (**Supplementary Figure 5**).

## 4 Discussion

The search of new drugs or alternative treatments for VL is an urgent goal for scientists and countries affected by this disease. In the present study, we aimed to evaluate the effect of NAC in *L. infantum* infection. A pilot clinical trial, using NAC as adjuvant to the standard VL treatment with meglumine antimoniate, shows that patients from the SbV + NAC group had higher sCD40L in sera during treatment, and these levels were negatively correlated with IL-10 levels. Data estimation show a negative correlation between the sCD40L levels and the spleen size and parasite load only in SbV + NAC group. However, the laboratorial and clinical parameters during disease evolution did not show any differences between the groups. *In vitro* experiments show that NAC treatment of stimulated PBMCs reduces the frequency of monocytes producing IL-10 and reduces the frequency of T CD4+ and CD8+ cells expressing inflammatory cytokines.

Arginase pathway in macrophages not only permits parasites to diminish the production of free radicals but also enhances the capacity of parasites to grow by the production of polyamines (Muxel et al., 2018). Moreover, dysregulated production of cytokines by immune response leads to unspecific activation of inflammatory cells that cause several tissues damage. Among the cytokines of higher importance in the VL pathogenesis, the production of IL-10, an important down regulatory cytokine of immune response, followed by production of pro-inflammatory cytokines as IL-6 and TNF-α, is associated to VL severity (Costa et al., 2013; dos Santos et al., 2016). Whereas, well-balanced secretion of sCD40L, IFN-γ, and IL-12 is associated to better clinical evolution in patients with VL (Badaro et al., 1990; Bacellar et al., 2000; de Oliveira et al., 2013). Therefore, the effect of NAC reduction of IL-10 *in vitro*—together with the demonstration that, in the pilot clinical trial described here, the NAC treatment induces a higher production of sCD40L and that this production negatively correlates with IL-10 levels—suggests that the effect of NAC is associated with a better regulation of the innate and adaptive immune responses. Although IL-10 is important to downregulate an exacerbated immune response, the presence of IL-10 in concomitance with pro-inflammatory cytokines leads to a non-specific and non-resolutive immune response and has been associated to worse clinical evolution (Gautam et al., 2011; Singh et al., 2021).

Unfortunately, the use of NAC as treatment adjuvant does not change clinical evolution and laboratorial parameter when compared to standard protocol. In further analysis, the sCD40L levels also negatively correlated with the spleen size in the SbV + NAC group, but not in the control SbV group. sCD40L has been previously demonstrated as an important biomarker of VL disease, associated with better outcomes, stimulating resolutive immune response (Okwor and Uzonna, 2016; Adriaensen et al., 2018). Previous data of our group, using patients from the same local of this study, demonstrate that sCD40L is an important biomarker in VL, indicating a favorable evolution during and after treatment, negatively correlating with the spleen sizes and parasite load in patients with VL (de Oliveira et al., 2013).

Previous data in experimental animals also demonstrated that BALB/c mice infected with different *Leishmania* species and treated with NAC have lower parasitism in footpad lesion without affect lesion swelling (Cruz et al., 2008; Monteiro et al., 2008), reduced oxidative stress in liver without diminished production of inflammatory profile (Gasparotto et al., 2017), and reduced oxidative stress associated to lower levels of cytokines and pain in infected mice (Crupi et al., 2020). These data are in concordance with results demonstrated in the present work, showing a variation in *in vivo* disease evolution, mixing enhanced response to parasite infection, but not always associated to a better evaluation in markers of disease.

In accordance with our results of the pilot clinical trial, the *in vitro* study described here shows that the addition of NAC in the presence or absence of meglumine antimoniate reduces the frequency of monocytes producing IL-10 without a change in frequency of TNF-α+ cells. The higher ratio of TNF-α/IL-10 in NAC-treated monocytes than that in non-treated cells suggests that NAC has an important role as a modulator of immune response, leading to the activation of microbicidal pathway of TNF-α. In contrast to this, the treatment with NAC in macrophage infected with *L. infantum* parasites does not reduce the parasite load. Our previous study has shown that sCD40L *in vitro* reduces the parasite numbers in macrophage cultures infected by *L. infantum* (de Oliveira et al., 2015). These data suggest that a combination of IL-10 downregulation and activation of CD40 pathway is needed. In addition to reducing the suppression of monocytes, NAC also reduces the presence of the effector T cells producing IL-2, TNF-α, and IFN-γ cytokines. These data suggest that NAC induces a resolutive immune response, especially when monocytes are in contact with lymphocytes, that could contribute to a better clinical evolution of patients with VL.

Although NAC could act as antioxidant donor to human cells and *Leishmania* parasites could be favored by antioxidant pathways in macrophage, the reduction of IL-10 might overcome the antioxidant effect. In addition, it is demonstrated that glutathione molecules, stimulated by NAC, could reduce activation of pro-inflammatory profile in human macrophages by inactivation of NF-κB transcription factor (Verhasselt et al., 1999; Fraternale et al., 2013). The use of glutathione donors decreases the production of TNF-α, IL-12, IL-6, IL-8, and IL-1β (Gosset et al., 1999; Haddad et al., 2001; Mazzeo et al., 2002), cytokines that are associated to the cytokine storm described in active VL, and some associated with worse prognosis, such as IL-6 and TNF-α. This mechanism of action together with our data is an evidence that NAC should be further tested as adjuvant in treatment of patients with VL, especially in the severe cases with cytokines storm. Therefore, on the basis of numerous studies in literature that demonstrate the importance of macrophage in the pathogenesis of VL disease and evolution (Liu and Uzonna, 2012), NAC as adjuvant therapy to antimony treatment might contribute to create a favorable immunological microenvironment in patients with VL. It is important to emphasize that the use of compound like NAC does not abrogate, but change the inflammatory response, making resolutive immune response and limiting the release of controversy cytokines, such as IL-10, and induces release of microbicidal agents as nitric oxide.

Although we have some positive results that demonstrate the effect of NAC in VL treatment, we could not demonstrate a clinical effect of this treatment, but there is no deleterious effect in any of the clinical parameters analyzed, demonstrating the safety of NAC even in association with antimony therapy. This study also did not observe the *in vitro* effect of NAC itself in the microbicidal activity of macrophages. Thus, more studies are needed, aiming to tests the use of NAC as adjuvant therapy in patients with VL, to compare its effect in different doses and in patients with different disease severities, and to elucidate *in vitro* mechanisms of action of NAC in *Leishmania* infection.

Together, the results obtained here show that NAC as adjuvant therapy of patients with VL is safe, has altered some aspects of the immune response, such as higher levels of sCD40L, with a strong negative correlation with IL-10 levels, and confirmed *in vitro* the control of IL-10 release by monocytes. These data suggest that NAC treatment might be a candidate to be further studied as adjuvant therapy to antimony for patients with VL.

## Data availability statement

The raw data supporting the conclusions of this article will be made available by the authors, without undue reservation.

## Ethics statement

The studies involving human participants were reviewed and approved by Comitê de Ética em Pesquisa da Universidade Federal de Sergipe. Written informed consent to participate in this study was provided by the participants’ legal guardian/next of kin.

## Author contributions

Recruitment of patients and clinical evaluation: EM, ND, AA, AS, and MOB. Performing *in vitro* experiments: LM, CS, MR, RS, FO, and PS. Data analysis: EM, LM, AJ, and RA. Scientifical revision and analysis: JS and ML. Conceptualization and supervision of all research: AJ and RA. Writing of the manuscript: LM, CS, ML, AJ, and RA.

## Funding

This work was supported by CHAMADA UNIVERSAL MCTI/CNPq n. 01/2016 (grant number: 429246/2016-1, RA) and by Universal, FAPITEC/SE, 2008. LM was sponsored by Postdoctoral Fellowship from INCT Imuno/CAPES. CS is sponsored by CAPES. JS, AJ, and RA are scientists supported by the Brazilian Research and Technology Council (CNPq). CS has Doctorate fellowship by CAPES.

## Acknowledgments

All authors are grateful to the participating patients of this study. We also recognize the contributions of the medical team in administration and clinical monitoring at HU-EBSERH/UFS and the researchers and students of LIBM from UFS for support in the experimental studies.

## Conflict of interest

The authors declare that the research was conducted in the absence of any commercial or financial relationships that could be construed as a potential conflict of interest.

The reviewer VA declared a shared affiliation with the author MR to the handling editor at the time of review.

## Publisher’s note

All claims expressed in this article are solely those of the authors and do not necessarily represent those of their affiliated organizations, or those of the publisher, the editors and the reviewers. Any product that may be evaluated in this article, or claim that may be made by its manufacturer, is not guaranteed or endorsed by the publisher.
