# Supplementary material for: Use of N-acetylcysteine as treatment adjuvant regulates immune response in visceral leishmaniasis: Pilot clinical trial and in vitro experiments
Source: Front Cell Infect Microbiol. 2022 Nov 24;12:1045668. doi: 10.3389/fcimb.2022.1045668 (PMC9730326; doi:10.3389/fcimb.2022.1045668)
Supplement: Supplementary file 1 [file DataSheet_1.pdf]

Supplementary Figure 1

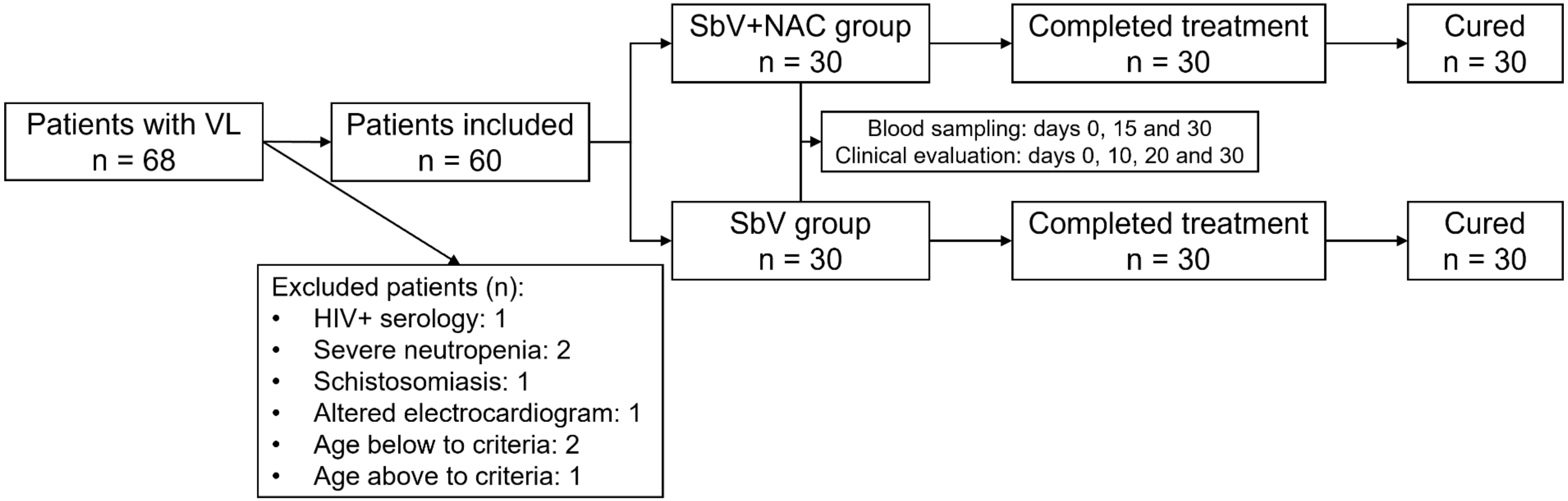

Flow diagram summarizing selection of patients, treatment randomization, biological and clinical sampling, and clinical outcomes.

Supplementary Table 01 Clinical and laboratorial data of patients diagnosed with VL and before leishmanicidal treatment (D0)

Variable	Test group (mean ± SD)	Control group (mean ± SD)	*P*
Age (years)	12.5 ± 11.1	13.2 ± 12.8	0.81
Male (%)	16 (53.3%)	19 (63.3%)	0.43
Liver (cm below RCM)	5.3 ± 3.6	5.4 ± 3.2	0.89
Spleen (cm below RCM)	10.5 ± 4.7	8.1 ± 4.5	0.09
Hemoglobin (g/dL)	8.9 ± 1.7	8.7 ± 1.3	0.55
Leucocytes (/mm3)	2684 ± 1076	3167 ± 1585	0.17
Neutrophils (/mm3)	861 ± 491	1062 ± 754	0.23
Lymphocytes (/mm3)	1387 ± 789	1632 ± 966	0.29
Eosinophils (/mm3)	37.5 ± 71.3	32.7 ± 47.2	0.91
Plaquettes (/mm3)	169700 ± 62652	174273 ± 64840	0.74
Creatinine (mg/dL)	0.54 ± 0.23	0.62 ± 0.27	0.19
Urea (UI/dL)	21.7 ± 6.8	24.4 ± 10.9	0.26
AST/TGO (UI/dL)	103.7 ± 234.9	125.5 ± 184.8	0.13
ALT/TGP (UI/dL)	64.7 ± 106.9	84.1 ± 99.9	0.23
γGT (UI/dL)	106.6 ± 152.1	155.4 ± 239.3	0.08
Amylase (UI/dL)	53.9 ± 52.9	60.1 ± 27.1	0.60

RCM: right costal margin
Test group: treated SbV+NAC; Control group: treated with SbV
Comparisons between groups were made using Mann-Whitney test

Supplementary Table 02 Clinical and laboratorial data of patients diagnosed with VL after experimental or standard treatments (D30)

Variable	Test group SbV+NAC	Control group SbV	*p*	Effect size
**Clinical parameters**				
Cure	30 (100%)	30 (100%)	> 0.99	1.00 (0.88; 1.12)
Liver (cm below RCM)	2.97 ± 2.50	2.85 ± 2.76	0.91	0.01
Spleen (cm below RCM)	5.87 ± 3.30	4.05 ± 3.34	0.07	0.27
**Laboratorial parameters**				
Hemoglobin (g/dL)	10.70 ± 1.13	10.78 ± 1.47	0.95	0.04
Leucocytes (/mm3)	5952 ± 2749	6644 ± 3259	0.54	0.08
Neutrophils (/mm3)	2278 ± 1168	2572 ± 1676	0.53	0.11
Eosinophils (/mm3)	498.3 ± 846.2	444.9 ± 484.6	0.88	0.01
Plaquettes (/mm3)	319880 ± 358726	289056 ± 83663	0.21	0.20
AST/TGO (UI/dL)	46.8 ± 12.77	49.65 ± 21.03	0.84	0.02
ALT/TGP (UI/dL)	45.42 ± 17.76	52.04 ± 23.96	0.38	0.12
**Immunological parameters**				
IL-10 (pg/mL)	9.48 ± 10.54	17.02 ± 35.34	0.94	0.01
TNF-α (pg/mL)	20.45 ± 12.92	45.54 ± 70.68	0.47	0.15
IL-12 (pg/mL)	10.32 ± 20.52	127.0 ± 350.2	0.13	0.31
sCD40L (pg/mL)	24530 ± 12759	9629 ± 13721	**0.003**	**0.58**

RCM: right costal margin
Test group: treated SbV+NAC; Control group: treated with SbV
For categorical variable: comparison was made using Fisher`s Exact test; Effect size: Relative Risk plus confidence interval 95%
For continuous variables: Mann-Whitney test was used; Effect size r: calculated using *Z* score from Mann-Whitney

Supplementary Table 03 Results of one-way multivariate analysis of variance (MANOVA) of age and group effects data and sCD40L effect between groups

Effect	Multivariate Tests[a]					
	**Statistic**	**Value**	**F**	**Sig.**	**Partial Eta Squared**	**Observed Power[c]**
**Age**	Pillai's Trace	0.013	0.093[b]	0.963	0.013	0.064
	Wilks' Lambda	0.987	0.093[b]	0.963	0.013	0.064
	Hotelling's Trace	0.013	0.093[b]	0.963	0.013	0064
	Roy's Largest Root	0.013	0.093[b]	0.963	0.013	0.064
**Group**	Pillai's Trace	0.425	5.184[b]	0.008	0.425	0.867
	Wilks' Lambda	0.575	5.184[b]	0.008	0.425	0.867
	Hotelling's Trace	0.741	5.184[b]	0.008	0.425	0.867
	Roy's Largest Root	0.741	5.184[b]	0.008	0.425	0.867
**Tests of Between-Subjects Effects**		Type III Sum of Squares				
**Corrected Model**	Rank of sCD40L D0	816.394[a]	4.309	0.026	0.273	0.691
	Rank of sCD40L D15	940.525[b]	5.914	0.008	0.340	0.829
	Rank of sCD40L D30	573.496[c]	7.419	0.003	0.392	0.907
**Group**	Rank of sCD40L D0	810.011	8.552	0.008	0.271	0.800
	Rank of sCD40L D15	913.628	11.489	0.003	0.333	0.901
	Rank of sCD40L D30	571.581	14.788	0.001	0.391	0.957

a. Design: Intercept + Age + Group
b. Exact statistic
c. Computed using alpha = .05
a. R Squared = .273 (Adjusted R Squared = .209)
b. R Squared = .340 (Adjusted R Squared = .282)
c. R Squared = .392 (Adjusted R Squared = .339)
d. Computed using alpha = .05

Supplementary Figure 02

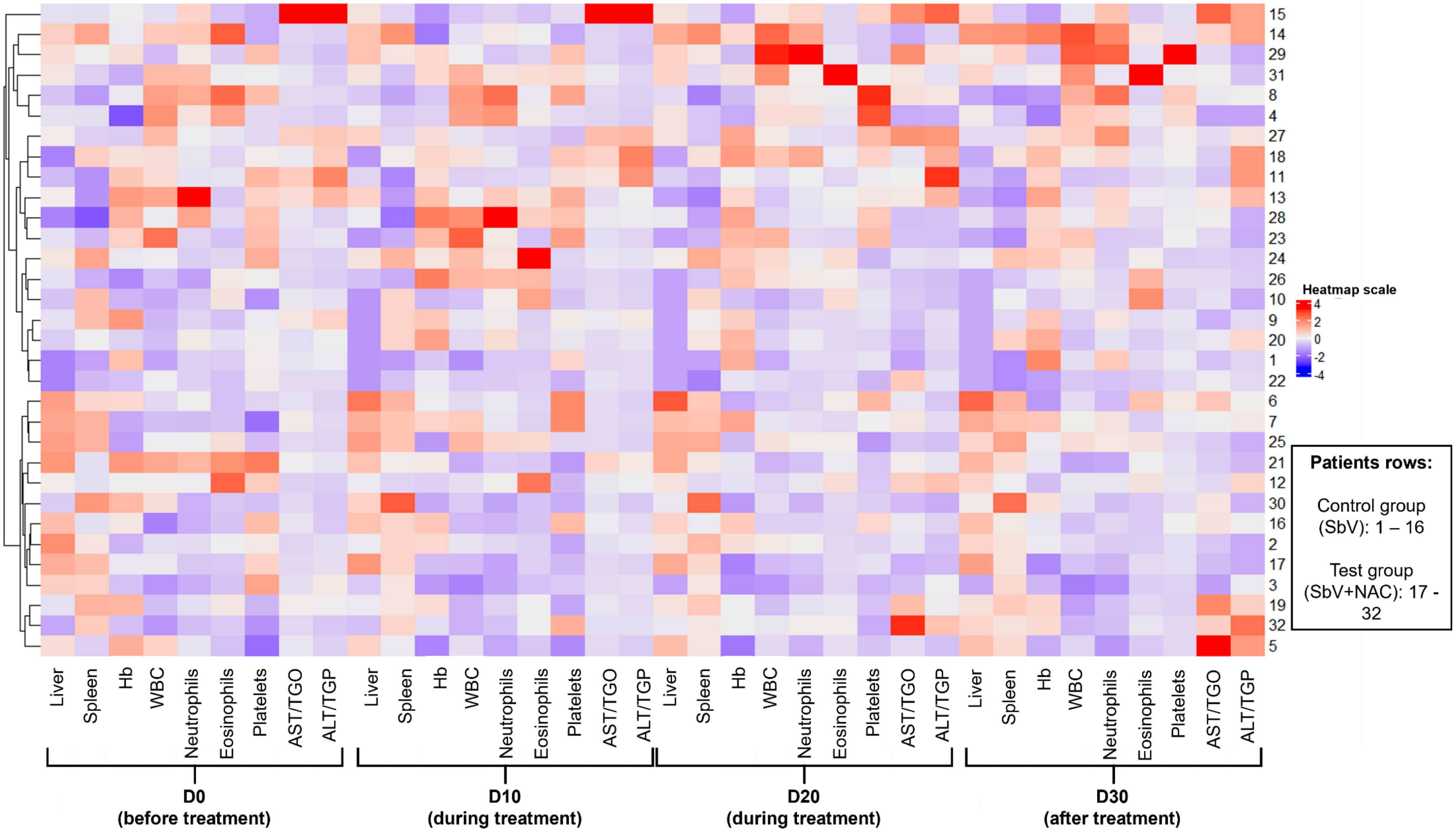

Cluster analysis and heatmap of distribution of clinical and laboratorial data of patients before, during and after leishmanicidal treatment in presence or absence of NAC. Heatmap was made using ComplexHeatmap package in R and Ward`s method (unsupervised hierarchical cluster analysis) was used to test if patients in Test or Control groups could be grouped *per si* based on data profile. The 32 patients (n = 16/group) were aleatory selected with no gap in data).

Supplementary Figure 03

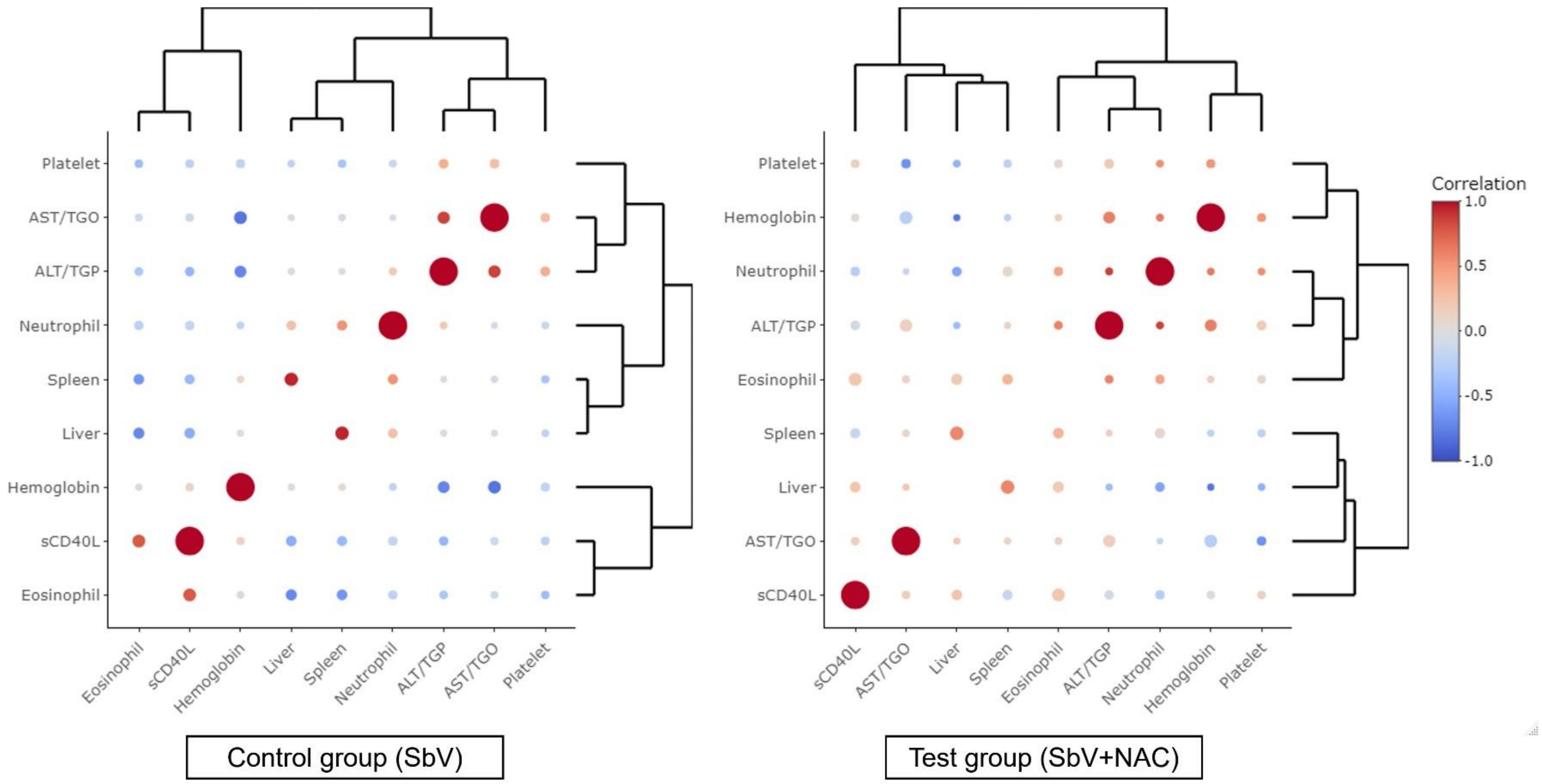

Correlation heatmap of clinical laboratorial data and sCD40L levels of patients during leishmanicidal treatment in presence or absence of NAC. Heatmap was made using Heatmaply package in R. Spearman correlation test was used to compare measures. Standard clusterization was selected.

Supplementary Figure 04

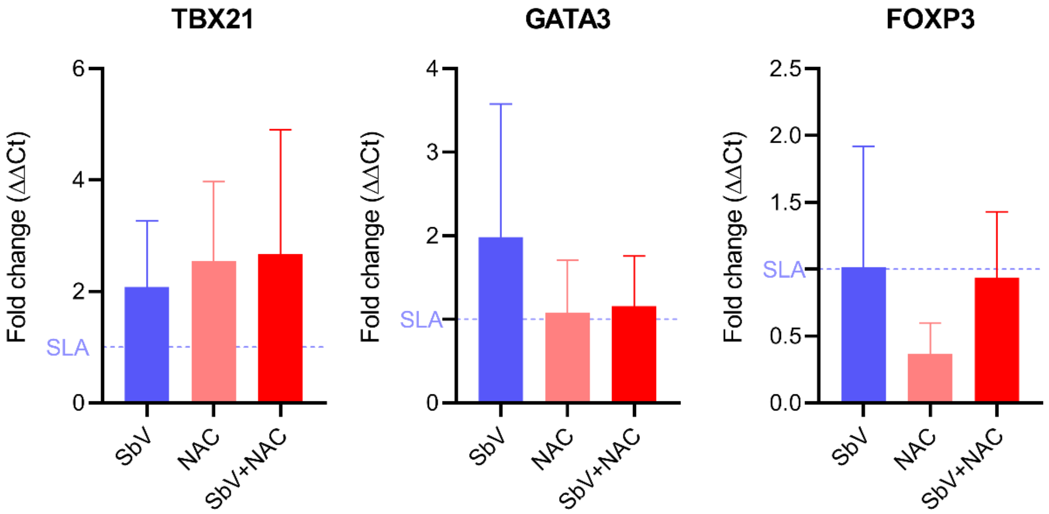

Analysis of mRNA expression in total PBMC stimulated with SLA and treated with SbV or NAC or SbV+NAC. The ΔΔCt was calculated first subtracting Unstimulated cells and after using SLA-nontreated cells as negative control. Bars represents Mean ± SEM from healthy donors (n = 4, aleatory selected from PBMC utilized to flow cytometry). Mann-whitney was used to compare groups.

Supplementary Figure 05

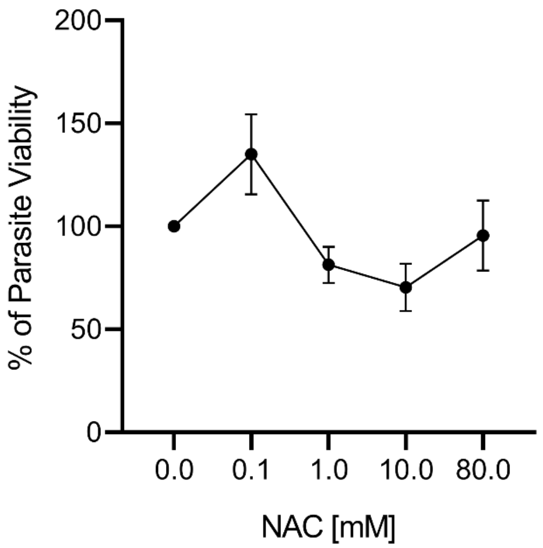

Survival of *L. (L.) infantum* promastigotes in increasing concentrations of NAC. Parasites were exposed for 48h, and motility was observed in Neubauer chamber. The initial concentration of parasites in the well was used as standard to parasite viability. Dots represents mean ± SEM of three independent experiments made in triplicate. Differences were calculated using Friedman followed by Dunn`s test. IC50: Half-maximal inhibitory concentration.
